# B-ALL With t(5;14)(q31;q32); *IGH-IL3* Rearrangement and Eosinophilia: A Comprehensive Analysis of a Peculiar *IGH*-Rearranged B-ALL

**DOI:** 10.3389/fonc.2019.01374

**Published:** 2019-12-10

**Authors:** Benjamin Fournier, Estelle Balducci, Nicolas Duployez, Emmanuelle Clappier, Wendy Cuccuini, Chloé Arfeuille, Aurélie Caye-Eude, Eric Delabesse, Elodie Bottollier-Lemallaz Colomb, Karin Nebral, Marie-Lorraine Chrétien, Coralie Derrieux, Aurélie Cabannes-Hamy, Florent Dumezy, Pascaline Etancelin, Odile Fenneteau, Jamile Frayfer, Antoine Gourmel, Marie Loosveld, Gérard Michel, Nathalie Nadal, Dominique Penther, Isabelle Tigaud, Elise Fournier, Bettina Reismüller, Andishe Attarbaschi, Marina Lafage-Pochitaloff, André Baruchel

**Affiliations:** ^1^Department of Pediatric Hematology and Immunology, University Hospital Robert Debré, Assistance Publique des Hôpitaux de Paris (APHP), Paris, France; ^2^Hematology Laboratory, University Hospital Paul-Brousse, Assistance Publique des Hôpitaux de Paris (APHP), Villejuif, France; ^3^Department of Hematology, University Hospital, Lille, France; ^4^Hematology Laboratory, University Hospital Saint-Louis, Assistance Publique des Hôpitaux de Paris (APHP), Paris, France; ^5^Department of Genetics, University Hospital Robert Debré, Assistance Publique des Hôpitaux de Paris (APHP), Paris, France; ^6^Department of Haematology, Institut Universitaire de Cancérologie de Toulouse, CHU de Toulouse, Toulouse, France; ^7^Department of Pediatric Oncology and Hematology, University Hospital of Dijon, Dijon, France; ^8^Children's Cancer Research Institute (CCRI), St. Anna Kinderkrebsforschung, Vienna, Austria; ^9^Department of Hematology, University Hospital of Dijon, Dijon, France; ^10^Hematology Laboratory, Hospital Saint Faron, Meaux, France; ^11^Teenagers and Young Adults Hematology Unit, University Hospital Saint-Louis, Assistance Publique des Hôpitaux de Paris (APHP), Paris, France; ^12^Department of Oncology Genetics, Henri Becquerel Center, Rouen, France; ^13^Hematology Laboratory, University Hospital Robert Debré, Assistance Publique des Hôpitaux de Paris (APHP), Paris, France; ^14^Department of Hematology, Hospital Saint Faron, Meaux, France; ^15^Department of Pediatric Oncology, Hematology, Immunology, University Hospital of Amiens, Amiens, France; ^16^Hematology Laboratory, Timone Hospital, Assistance Publique-Hôpitaux de Marseille (APHM), CNRS, INSERM, Centre d'Immunologie de Marseille-Luminy (CIML), Aix Marseille University, Marseille, France; ^17^Department of Pediatric Hematology, Aix Marseille University, Marseille, France; ^18^Laboratory of Cytogenetics, University Hospital of Dijon, Dijon, France; ^19^Department of Cytogenetics, Lyon-Sud Hospital, Lyon, France; ^20^Department of Pediatric Hematology and Oncology, St. Anna Children's Hospital, Department of Pediatrics and Adolescent Medicine, Medical University of Vienna, Vienna, Austria; ^21^Hematological Cytogenetics Laboratory, Timone Hospital-Assistance Publique-Hôpitaux de Marseille (AP-HM), Aix-Marseille University, Groupe Francophone de Cytogénétique Hématologique (GFCH), Marseille, France; ^22^Institut Universitaire d'Hématologie, EA-3518, University Hospital Saint-Louis, Assistance Publique des Hôpitaux de Paris (APHP), Paris, France

**Keywords:** acute lymphoblastic leukemia, cytogenetics and molecular genetics, clinical and molecular epidemiology, eosinophilia, *IKZF1* rearrangement

## Abstract

**Background:** B-cell acute lymphoblastic leukemia associated with t(5;14)(q31;q32); *IGH-IL3* is an exceptional cause of eosinophilia. The *IGH* enhancer on 14q32 is juxtaposed to the *IL3* gene on 5q31, leading to interleukin-3 overproduction and release of mature eosinophils in the blood. Clinical, biological and outcome data are extremely scarce in the literature. Except for eosinophilia, no relevant common feature has been highlighted in these patients. However, it has been classified as a distinct entity in the World Health Organization classification.

**Cases Presentation:** Eight patients with t(5;14)(q31;q32) treated by French or Austrian protocols were retrospectively enrolled. Array comparative genomic hybridization, multiplex ligation-dependent probe amplification or genomic PCR search for *IKZF1* deletion were performed in 7. Sixteen patients found through an exhaustive search in the literature were also analyzed.

For those 24 patients, median age at diagnosis is 14.3 years with a male predominance (male to female ratio = 5). Eosinophilia-related symptoms are common (neurologic in 26%, thromboembolic in 26% or pulmonary in 50%). Median white blood cells count is high (72 × 10^9^/L) and linked to eosinophilia (median: 32 × 10^9^/L). Peripheral blasts are present at a low level or absent (median: 0 × 10^9^/L; range: 0–37 × 10^9^/L). Bone marrow morphology is marked by a low blast infiltration (median: 42%). We found an *IKZF1* deletion in 5 out of 7 analyzable patients Outcome data are available for 14 patients (median follow-up: 28 months): 8 died and 6 are alive in complete remission.

Some of these features are concordant with those seen in patients with other *IGH*-rearranged B-cell acute lymphoblastic leukemias: young age at onset, male sex, low blast count, high incidence of *IKZF1* deletion and intermediate prognosis.

**Conclusion:** Based on shared epidemiological and biological features, B-cell acute lymphoblastic leukemia with t(5;14)(q31;q32) is a peculiar subset of *IGH*-rearranged B-cell acute lymphoblastic leukemia with an intermediate prognosis and particular clinical features related to eosinophilia.

## Background

B-cell acute lymphoblastic leukemia (B-ALL) with translocation t(5;14)(q31;q32); *IGH-IL3* is an exceptional cause of massive eosinophilia recognized by the World Health Organization (WHO) classification of hematological malignancies as a distinct entity (1, 2). The translocation t(5;14)(q31;q32) juxtaposes the *IGH* enhancer located on 14q32 to the *IL3* gene on 5q31. The subsequent production of interleukin-3 (IL3) induces the maturation and release of eosinophils in the blood stream (3–5). The relationship between IL3 and eosinophilia is also supported by the constant eosinophilia in all patients treated with recombinant IL3 for Blackfan-Diamond anemia in one study (6). The IL3-receptor has been hypothesized to be present on blast cells and to induce JAK-STAT-dependent cell survival and proliferation in an autocrine manner (4, 7). The other part of the translocation involves the *IGH* gene, which is frequently rearranged via chromosomal translocations in mature B-cell neoplasms such as Burkitt leukemia/lymphoma, mantle cell or follicular lymphomas with *MYC, CCND1*, or *BCL2* as partner genes, respectively, whose expression is upregulated (8). *IGH* translocations are also reported in B-ALL and usually involve other partners such as members of the *CEBP* gene family, *ID4, CRLF2* or *EPOR* (9–13). Due to the rarity of t(5;14)(q31;q32) associated B-ALL, little is known regarding clinical and molecular features. To further characterize this very rare entity, we have analyzed in detail 8 new patients and combined these data to those of 16 patients from the literature over 35 years.

## Methods

### Patients

Patient #1 and patient #2 were first diagnosed in two of our centers in the same period. Consequently, we screened the database of FRALLE 2000 French ALL treatment study (2000–2010) and launched a call within the GFCH (Groupe Francophone de Cytogénétique Hématologique) for patients with B-ALL presenting with t(5;14)(q31;q32). Four other cases were retrieved including a recently relapsed case previously reported at diagnosis (14) and one published very recently (15). All these 6 patients were diagnosed between 2004 and 2016 and were treated according to national protocols (FRALLE 2000 for the 4 pediatric patients, GRAALL 05 and 14 for the 2 adult patients). Given the rarity of the disease, we seized the opportunity to include 2 additional patients diagnosed and treated in Austria in 1999 and 2010 according to the Associazione Italiana di Ematologia Oncologia Pediatrica and the Berlin-Frankfurt-Münster Acute Lymphoblastic Leukemia 2000 [AIEOP-BFM ALL 2000 (16)] and 2009 protocols.

### Conventional Cytogenetics

Karyotypes were analyzed on bone marrow samples at diagnosis and relapse according to the International System for Human Cytogenetic Nomenclature (ISCN, 2016). Whenever possible, at least 20 metaphases were analyzed.

### Fluorescence *in situ* Hybridization

Fluorescence *in situ* hybridization (FISH) was performed on cytogenetic preparations using an *IGH* dual-color break-apart probe (Vysis, Abbott Molecular Inc.) according to the manufacturer's instructions.

### Molecular Genetics

*IKZF1* deletions were detected using both multiplex ligation-dependent probe amplification (MLPA) (P335-B2 kit; MRC Holland) and genomic fluorescent multiplex PCR (17). MRD monitoring was based on *Ig-TCR* rearrangements (18, 19) except in patient #4 (*IGH-IL3* genomic PCR quantification).

### Array-Based Comparative Genomic Hybridization (a-CGH) and SNP-Array Analysis

a-CGH (SurePrint G3 Human CGH Microarray Kit, 4 × 180K; Agilent Technologies, USA) or SNP-array (CytoScan™ HD array; ThermoFisher Scientific) was performed in all cases (except for patient #8) according to manufacturers' instructions. Data were analyzed with the DNA Analytics software v4.0.85 and the Chromosome Analysis Suite version 3.1 (ChAS; Affymetrix) software package and annotated using the human genome version 19 (hg19) of the UCSC Genome Browser, excluding copy number variants (CNVs) arising from B-cell and T-cell antigen receptor gene rearrangements and known CNVs reported in public databases.

### Informed Consent

The study was approved by the local ethics committee (Comité Local d'Ethique pour la recherche clinique des HUPSSD Avicenne-Jean Verdier-René Muret (CLEA), December 13th, 2018, CLEA-2018-58). According to French legislation, due to the retrospective observational design of the study, informed consent from patient was waived.

## Cases Presentation

Firstly, we report here on our 8 patients selected for the presence of the specific t(5;14)(q31;q32). Clinical characteristics are detailed in Table 1, main biological features in Table 2 and outcome in Table S1. Patients #3 and #7 were previously reported (14, 15), but are yet further described here because of availability of new relevant biological data and because of occurrence of events during longer follow-up.

**Table 1:** Clinical characteristics at onset of the 8 newly reported patients.

	**Pt #1**	**Pt #2**	**Pt #3**	**Pt #4**	**Pt #5**	**Pt #6**	**Pt #7**	**Pt #8**
**Gender**	**Male**	**Male**	**Female**	**Male**	**Male**	**Male**	**Female**	**Male**
Age at diagnosis (years)	11	18	14	14	39	31	10	8
General symptoms	Fever, night sweats	Night sweats, asthenia, anorexia	Asthenia	Absent	Absent	Absent	Absent	Fever, asthenia
Organomegaly	HMG, SMG	Absent	Absent	Absent	Absent	SMG	HMG, SMG	HMG
Lung involvement	Dyspnea Chest X-ray: bilateral interstitial infiltrates	Absent	Dyspnea	Absent	Absent	Absent	Cough	Absent
Thrombo-embolic event	Absent	Absent	Absent	Absent	Multiple venous thrombosis	Lower limb venous thrombosis, pulmonary embolism	Absent	Absent
Cardiac involvement	Absent	Absent	Absent	Absent	Absent	Absent	Absent	Loeffler endocarditis
Neurologic involvement	Pyramidal syndrome, hemiparesia, acute confusional state	Absent	Absent	Pyramidal syndrome, hemiparesia, acute confusional state	Seizure, transient stroke, acute confusional state	Absent	Absent	Absent
CNS status	CNS1	CNS1	CNS1	CNS1	CNS1	CNS1	CNS1	CNS1
Skin involvement	Absent	Absent	Absent	Erythematous rash, pruritus	Erythematous rash	Absent	Erythematous rash	Absent

*Pt, Patient; HMG, Hepatomegaly; SMG, Splenomegaly; NS, Not specified*.

*CNS status: CNS1, non-traumatic lumbar punction with no blast on cerebrospinal fluid after centrifugation*.

**Table 2:** Biological characteristics at onset of the 8 newly reported patients.

	**Pt #1**	**Pt #2**	**Pt #3**	**Pt #4**	**Pt #5**	**Pt #6**	**Pt #7**	**Pt #8**
**Peripheral blood**								
White blood cells (×10^9^/L)	125	114	41	72	15.9	21.1	73.1	54.8
Eosinophils (×10^9^/L)	62.3	96	31.2	50	6	7.9	17.5	32.3
Basophils (×10^9^/L)	4.9	5.7	0.3	0.7	0.7	0.07	0	2.7
Neutrophils (×10^9^/L)	24.9	5.7	NS	14	6	1.9	8.8	14.3
Lymphocytes (×10^9^/L)	6.2	4	NS	5	NS	2.7	9.5	5.5
Monocytes (×10^9^/L)	1.3	0	NS	1.4	NS	0.5	0	0
Hemoglobin (g/dl)	13.5	12	13.4	11.3	12	12.1	12.3	8.9
Platelets (×10^9^/L)	310	320	309	387	259	207	70	313
Blasts (×10^9^/L)	18.7	2	0	0	0.2	0	36.6	0
**Bone marrow morphology**								
Blasts (%)	68.5	80	20	19	62	32	78	5
Eosinophils (%)	10.5	10	32	35	18	24	7	34
Basophils (%)	1.5	0	NS	1	0	1	NS	NS
**Immunophenotypic features**								
Immunophenotype (according to EGIL classification)	B-III	B-II	B-III	B-II	B-II	B-II	B-II	B-II
Myeloid markers (CD13+, CD33+)	–	–	CD33+	–	CD33+	–	NS	–

*Pt, Patient; NS, Not specified*.

We chose to highlight hereunder the main clinical features of these patients according to age at onset: pediatric/adolescent onset (8–18 years old) and young adult onset (21–39 years old). We then analyzed them together with the 16 patients from the literature in a third section.

### Pediatric Onset

Six patients were children or adolescent (8, 10, 11, 14, 14, and 18 year-old) including one with Down syndrome (patient #7). Two patients presented severe neurologic involvement (encephalopathy with pyramidal syndrome and acute confusion). Magnetic resonance imaging (MRI) showed scattered involvement of white matter and myelitis in one case, multiple cortical and subcortical ischemic lesions in another. Symptoms disappeared after the first courses of chemotherapy. No thrombo-embolic event was recorded. Peripheral blast cells count was increased in 2 (36.6 and 18.7 × 10^9^/L) but was low in others (range: 0–2 × 10^9^/L) and even absent in 10. Peripheral eosinophilia was increased (range: 17.5–96 × 10^9^/L). All patients reached complete remission after induction therapy. All patients are in first (*n* = 3), second (*n* = 1), or third (*n* = 1) continuous complete remission (CCR) for more than 3 years, except for the patient with Down syndrome who died from disease progression after a second relapse.

### Young Adult Onset

Two patients were young adults (31 and 39 years of age). Both presented with severe thrombo-embolic events (lower limb venous thrombosis with pulmonary embolism and multiple venous thrombosis with transient strokes, respectively). No encephalopathy was documented. Multiple but asymptomatic subcortical lesions on MRI were present in 1 patient.

Peripheral blast cells count was 0 and 0.2 × 10^9^/L, respectively. Eosinophilia was much lower (7.9 and 6 × 10^9^/L). Prognosis was poor: none reached complete remission after induction therapy, one is in second complete remission (CR2) 1 month after hematopoietic stem cell transplantation and one is deceased after relapse.

### Combined Analysis with Patients From The Literature

We next pooled and analyzed our 8 newly described patients with 16 patients found through an extensive search in the literature, to enlighten the specific features of this very rare entity (20–35). Consequently, the following data concern 24 patients (recognizing nevertheless that not all features are constantly available for each patient of the literature) (Table 3).

**Table 3:** Main characteristics of 24 patients at onset of t(5;14)(q31;q32) B-ALL -our patients (*n* = 8) and patients from literature (*n* = 16).

Clinical presentation^*^
Age (years) (median) [range]	14.3 [4–60]
Sex ratio (male/female)	20/4 (5)
Organomegaly	10/19 (53%)
Lung involvement	10/20 (50%)
Thrombo-embolic event	5/19 (26%)
Cardiac involvement	9/19 (47%)
Neurologic involvement	5/19 (26%)
Skin involvement	5/19 (26%)
**Peripheral blood (median) [range]**	
White blood cells (×10^9^/L)	72 [15.9–148.3]
Eosinophils (×10^9^/L)	31.7 [6–111]
Basophils (×10^9^/L)	0.8 [0–7]
Neutrophils (×10^9^/L)	9.7 [1.4–27.4]
Lymphocytes (×10^9^/L)	5.8 [2.7–13.1]
Monocytes (×10^9^/L)	0.9 [0.45–2.8]
Hemoglobin (g/dL)	12 [7–13.7]
Platelet (×10^9^/L)	171 [60–532]
Blasts (×10^9^/L)	0 [0–36.6]
**Bone marrow morphology (median) [range]**	
Blasts (%)	42 [5–80]^**^
Eosinophils (%)	24 [7–53]
Basophils (%)	1 [0–1.5]
Immunophenotypic features^*^	
Immunophenotype (EGIL classification)	1/12 BI 7/12 BII 4/12 BIII
Myeloid markers (CD13+, CD33+)	4/13 (31%)
**Cytogenetic features (median) [range]**	
Abnormal metaphases (%)	20 [3–100]
Additional cytogenetic abnormalities (%)	9/24 (37.5%)
Response to treatment^*^	
Complete remission after induction therapy	9/14
Relapse	9/14

* *The variable number of evaluable patients in the literature according to the different items explains a denominator <24*.

** *The presence of the t(5;14)(q31;q32) allows diagnosis of cases with <20% blast cells according to WHO classification*.

Median age at onset was 14.3 years (range: 4–60 years); median age for pediatric (*n* = 16) and adult patients over 18-year-old (*n* = 8) was 11 and 32 years, respectively. Nineteen out of 24 patients (79%) were above 10 years of age.

A strong male predominance was observed (20 males vs. 4 females).

Eosinophilia-related symptoms were common at onset. Ten out of 20 (50%) patients had lung involvement (dyspnea, cough, chest pain, pleural effusions with eosinophilia, interstitial alterations on chest X-ray). Four patients presented multiple venous thromboses (pulmonary embolism in 3 patients and cerebral venous thrombosis in 1), 1 patient had arterial thrombosis of the lower limb [i.e., 5 out of 19 (26%)]. Nine out of 19 (47%) patients had cardiac involvement, e.g., newly acquired ECG abnormalities, global cardiac failure (*n* = 1), Loeffler myocarditis on MRI (*n* = 3), restrictive cardiomyopathy (*n* = 1), or ventricular hypertrophy (*n* = 1), leading to death due to ventricular fibrillation in one patient. Five out of 19 (26%) patients had neurologic symptoms (decreased consciousness, cerebral palsy, generalized seizure, and transient ischemic attack). MRI data were available for 3 of our patients (described in text above) and in Kobayashi et al. (34). No patient had blasts in the cerebrospinal fluid at initial presentation. Five out of 19 (26%) patients had an erythematous urticaria-like skin involvement.

Regarding biologic parameters, median white blood cell count was high (72 × 10^9^/L), mainly due to eosinophilia (median: 32 × 10^9^/L; range: 6–111 × 10^9^/L). Basophil count was increased (median: 0.8 × 10^9^/L; range: 0–7 × 10^9^/L). Hemoglobin and platelets were mainly normal (median: 12 g/dL, range 7–13.7 g/dL, and 171 × 10^9^/L, range 60–532 × 10^9^/L, respectively). Peripheral blasts were present at a very low count or even absent in 9 patients (median: 0 × 10^9^/L; range 0–36.6 × 10^9^/L). Of note, in these 9 patients, blast count was determined only on blood smears except for 2 patients who had flow cytometry analysis [patient #6 and reference (35)]. An only partial lymphoblastic infiltration in the bone marrow was frequent (median 42%; range: 5–80%). Two patients had a prolonged interval between blood eosinophilia with normal bone marrow morphology and the appearance of blast cells in the bone marrow [12 months for patient #5, 6 months in Knuutila et al. (26)].

Flow cytometry data were available for 12 patients classified patients in BI (*n* = 1), BII (*n* = 7), or BIII (*n* = 4) subsets according to EGIL classification. Myeloid markers (CD13 and/or CD33) were positive in 4/13 patients (31%).

Regarding cytogenetics, detailed karyotypes are provided in Table S2 and array-based Comparative Genomic Hybridization (a-CGH) in Table S3. All but 2 patients had an abnormal karyotype harboring the t(5;14)(q31;q32): concerning patient #6 and in Yu et al. (35), the translocation was proven by genomic *IGH-IL3* PCR or next generation sequencing, respectively. t(5;14)(q31;q32) was the sole cytogenetic finding in 13 patients, 9 having additional abnormalities. The number of abnormal metaphases was low (range: 1–22), except for one case where all metaphases were abnormal.

a-CGH abnormalities found in at least 2 patients included deletion of *SLX4IP* (*n* = 4), *VPREB1* (*n* = 3), *CDKN2A* (*n* = 3), *PAX5* (*n* = 2), *MEF2C* (*n* = 2), *ARHGAP24* (*n* = 2), and *ETV6* (*n* = 2).

An *IKZF1* deletion was searched only in our newly described patients and detected in a large proportion [5/7 (71%), Table S4]. The isochromosome 7q in Knuutila et al. (26) also leads to the loss of *IKZF1*.

Outcome and remission status were both available for 14 out of 24 patients [Figure S1: our 8 patients (detailed in Table S1), 6 from literature (20–22, 28, 30) with additional information provided by Toboso and Campos (33)]. Median follow-up was 28 months (range: 4 days−13 years). Early treatment response was characterized by a high induction failure proportion: only 9 out 14 patients were in complete remission (CR) after first induction, 4 more reached CR1 after a further course. Minimal residual disease (MRD), only available for our newly described patients, was high for 4 patients after first induction (range: 0.1–10%). Eight out of 14 patients died [cardiac involvement secondary to eosinophilia (*n* = 1) (20), uncontrolled disease and/or sepsis (*n* = 7)].

## Discussion

t(5;14)(q31;q32); *IGH-IL3* B-ALL is an exceptional hence poorly described subset of B-ALL, which is however described as a distinct entity in the WHO classification. None was reported despite the use of extensive FISH screening for *IGH*-translocation in 3,302 patients from UK protocols (9). By combining 8 new patients (spanning over 15 years of French and Austrian protocols) to 16 patients from the literature (20–35), we could highlight new striking features.

(1) Most patients are adolescents or young adults (median age: 14.3 years). (2) Male to female ratio is highly biased (5 to 1). (3) Eosinophilia-related symptoms are common and severe. It is the expected consequence of the underlying translocation: IL-3 overproduction induces maturation and release of eosinophils in the blood from the bone marrow. (4) Lymphoblastic infiltration of the bone marrow is partial and explains that peripheral blasts are almost undetectable in a vast proportion of patients. This partial infiltration may also account for normal to slightly decreased hemoglobin and platelet count. In cases with <20% of infiltrating blast cells, the specific translocation permitted a diagnosis of t(5;14)(q31;q32) associated B-ALL (2). The frequent CNS-related symptoms with no ALL blast infiltration suggests a direct eosinophil toxicity on neuronal, glial and endothelial cells caused either by eosinophilic proteins release or eosinophil cells infiltration (36, 37). Depending on the damaged cell type, symptoms may include signs of encephalopathy or focal symptoms due to local thrombosis and multiple infarcts (38). (5) *IKZF1* deletions are frequently detected. (6) Response to treatment is poor: induction failure and high levels of MRD are frequently observed, and may account for the intermediate prognosis.

These particularities suggest new diagnostic strategies, notably because (a) peripheral blasts are often absent (even by flow cytometry analysis) and (b) the translocation t(5;14)(q31;q32) may be missed owing to the low rate of abnormal metaphases on standard karyotype and metaphase FISH. It is worth noting that the 2 patients with a prolonged interval between blood eosinophilia and the appearance of blast cells in the bone marrow could fulfill criteria of idiopathic hypereosinophilic syndrome (39). Persistent and/or massive eosinophilia should thus be investigated by repeated bone marrow aspirations, including cytogenetic investigations and *IGH*-FISH on cytogenetic pellet on a sufficient number of metaphases and interphase nuclei. FISH analysis on bone marrow smears (patients #2 and #6) or on leukemic cytometer-sorted cells (34) (patient #8) can be used. As suggested in (35, 40), a bone marrow biopsy may reveal focal clusters of blasts missed by bone marrow aspiration. Next generation sequencing may be an alternative to detect *IGH-IL3* rearrangement (35)_._ Finally, careful monitoring of eosinophils should be performed during and after treatment as increased eosinophils may suggest a relapse.

Of note, t(5;14)(q31;q32); *IGH-IL3* B-ALL shares remarkable features with other *IGH*-rearranged B-ALL (5–11% of B-ALL) (9, 10, 12, 41) (Table 4). (1) *IGH*-rearranged B-ALL also affects especially adolescents and young adults [median age of onset: 25 years (12) or 16 years (9)]. (2) A male predominance is present even if male to female ratio is lower (1.44) (12). (3) In both entities, a majority of patients with low blast count is observed (9, 10, 41). (4) A high incidence of *IKZF1* deletion (40%) is frequent in patients with *IGH*-rearranged B-ALL (9). (5) Only 6 out 14 evaluable patients with t(5;14)(q31;q32); *IGH-IL3* B-ALL are alive which mirrors the 27–30% overall survival observed in 3 studies (9, 12, 41).

**Table 4:** Comparison of *IGH*-rearranged B-ALL from literature to t(5;14)(q31;q32); *IGH-IL3* B-ALL (our series plus published cases).

		*IGH* patients^*^	***IGH-IL3* patients**	**Age at diagnosis (median) [range]**	**Male to female ratio**	**White blood cells (x10^**9**^/L)**	***IKZF1* deletion**	**Additional cytogenetic abnormalities**
This study^**^	All patients	24	24/24 (100%)	14.3 years [4–60 years]	20/4	<50: 9/21 (43%) >50: 12/21 (57%)	5/7 (71%)	9/23 (39%)
	0–19 years-old	17	17/17 (100%)	11 years [4–19 years]	14/3	<50: 3/17 (18%) >50: 14/17 (82%)	3/4 (75%)	4/18 (22%)
	20–60 years-old	7	7/7 (100%)	33 years [22–60 years]	6/1	<50: 6/6 (100%) >50: 0/6 (0%)	2/2 (100%)	5/7 (71%)
Russell et al. (9)	UKALL2003	103/2,572 (4%)	0/2,572 (0%)	5 years [1–24 years]	1.6/1	<50: 80/103 (78%) >50: 23/103 (22%)	21/58 (36%)	89/143 (62%)
	UKALLXII	56/697 (8%)	0/697 (0%)	33 years [15–64 years]	1.2/1	<50: 39/55 (71%) >50: 16/55 (29%)	14/30 (47%)	
Chapiro et al. (12)		29/4,362 (0.6%)	0/4,362 (0%)	25 years [5–85 years]	1.4/1	NS	NS	19/29 (66%)
Lafage-Pochitaloff et al. (41)		27/542 (5%)	0/542 (0%)	43 years [18–59 years]	0.6/1	<30: 23/27 (85%) >30: 4/27 (15%)	NS	22/27 (81%)

* *Incidence as determined by IGH FISH and/or 14q32 translocations*.

** *Our series (n = 8) plus published cases (n = 16)*.

*Some data were not evaluable for all published cases, explaining a variable denominator*.

*5y EFS, 5-year event-free survival; 5y OS, 5-year overall survival; CI, Confidence interval; NS, Not specified*.

In conclusion, t(5;14)(q31;q32)*; IGH-IL3* B-ALL constitutes a very rare subset of *IGH*-rearranged B-ALL. The *IL3* partner gene is responsible for the specific symptomatology and increased awareness of this B-ALL may lead to a correct diagnosis of B-ALL when facing a severe symptomatic eosinophilia. International collaboration should provide new insights into its biology and bring new treatment strategies.

## Data Availability Statement

The datasets generated for this study are available on request to the corresponding author.

## Ethics Statement

The studies involving human participants were reviewed and approved by Comité Local d'Ethique pour la recherche clinique des HUPSSD Avicenne-Jean Verdier-René Muret (CLEA), December 13th, 2018, CLEA-2018-58. Written informed consent from the participants' legal guardian/next of kin was not required to participate in this study in accordance with the national legislation and the institutional requirements.

## Author Contributions

BF, EB, AA, ML-P, and AB: conception of the study. All authors: collection and assembly of data, final approval of manuscript, and accountable for all aspects of the work. BF, EB, ML-P, and AB: data analysis and interpretation and manuscript writing.

### Conflict of Interest

The authors declare that the research was conducted in the absence of any commercial or financial relationships that could be construed as a potential conflict of interest.
