# Supplementary material for: B-ALL With t(5;14)(q31;q32); IGH-IL3 Rearrangement and Eosinophilia: A Comprehensive Analysis of a Peculiar IGH-Rearranged B-ALL
Source: Front Oncol. 2019 Dec 10;9:1374. doi: 10.3389/fonc.2019.01374 (PMC6914849; doi:10.3389/fonc.2019.01374)
Supplement: Supplementary file 1 [file Table_1.DOCX]

| **Table S1.** Outcome of the 8 newly reported patients.  Pt, patient; CNS: Central nervous system; CR, complete remission; CCR, continuous complete remission;  HSCT: Hematopoietic Stem Cell Transplantation; BMT: Bone Marrow Transplantation; MUD, matched related donor; UCB, unrelated cord blood transplantation  NA, not available.  ^(1)^ Chemosensitivity at day 21: M1 marrow ie. blasts < 5% on bone marrow aspiration at day 21 of induction therapy  ^(2)^ Complete remission: blasts < 5% in bone marrow after induction therapy  ^(3)^ MRD, minimal residual disease; MRD was determined using Ig-TCR clone-specific probes except in Pt # 4 where an *IGH-IL3* specific probe was used. | | | | | | | | |
| --- | --- | --- | --- | --- | --- | --- | --- | --- |
|  | **Pt #1** | **Pt #2** | **Pt #3** | **Pt #4** | **Pt #5** | **Pt #6** | **Pt #7** | **Pt #8** |
| **Treatment protocol** | FRALLE 2000  High risk | FRALLE 2000  High risk | FRALLE 2000  High risk | FRALLE 2000  High risk | GRAALL 2005 | GRAALL 2014 | ALL-BFM 99 pilot  Low risk | AIEOP-BFM ALL 2009  High risk |
| **Chemosensitivity**  **at day 21^(1)^** | Yes | No | Yes | Yes | NA | No | Yes | Yes |
| **Complete remission after induction therapy^(2)^** | Yes | Yes | Yes | Yes | No | No | Yes | Yes |
| **MRD after**  **induction therapy**^(3)^ | Negative | 0.5% | 0.7% | Negative | NA | 10% | Low positive, unquantifiable | 0.1% |
| **MRD at the start of delayed intensification therapy** | Negative | 0.5% | 0.1% | Negative | NA | NA | Negative | 0.01% |
| **HSCT** | No | Geno-identical BMT in CR1 | UCB 4/6  In CR2 | No | Geno-identical BMT  in CR1 | Geno-identical BMT  in CR2 | No | MUD in CR2 and CR3 |
| **Relapse after CR1** | No | No | Isolated medullary relapse  10 years from CR1 | No | Isolated CNS relapse (CNS3)  14 months from CR1 | Relapse  4 months from CR1 | Isolated medullary relapse  6 years from CR1 | Isolated medullary relapse  3 years from CR1 |
| **t(5;14)(q31;q32) at relapse** | Not applicable | Not applicable | Microdeletion next to *IL3* gene | Not applicable | NA | Yes | Yes | Yes |
| **Outcome** | CCR1  36 months + | CCR1  38 months + | CCR2  39 months + | CCR1  48 months + | Death  12 months after relapse | Under treatment | Death  3 years from diagnosis | CCR3  38 months + |
|  | | | | | | | | |
