# Supplementary material for: B-ALL With t(5;14)(q31;q32); IGH-IL3 Rearrangement and Eosinophilia: A Comprehensive Analysis of a Peculiar IGH-Rearranged B-ALL
Source: Front Oncol. 2019 Dec 10;9:1374. doi: 10.3389/fonc.2019.01374 (PMC6914849; doi:10.3389/fonc.2019.01374)
Supplement: Supplementary file 2 [file Table_2.DOCX]

| **Table S2**. Detailed karyotypes at diagnosis of the 24 B-ALL patients with t(5;14)(q31;q32) including our patients (n=8)  [ ] : when available the number of metaphase cells is specified in [ ]; NA: not applicable; ND: not done; Pt: Patient  *: t(5;14) confirmed on a subsequent karyotype | | | | |
| --- | --- | --- | --- | --- |
| **Age**  **(years)** | **Karyotype** | **Abnormal metaphases (%)** | ***IGH* break apart FISH** | **Reference** |
| 5 | 46,XX,t(5;14)(q31;q32)[13]/46,XX[2] | 87 | ND | Chen et al, 1992 |
| 6 | 46,XY,t(5;14)(q31;q32)[2]/46,XY[17] | 11 | ND | Delabesse et al, 1995 |
| 7 | 46,XY,t(5;14)(q31.1;q32.3)[15]/46,XY[4] | 79 | ND | Baumgarten et al, 1987 |
| 8 | 46,XY,t(5;14)(q31;q32)[3]/46,XY[17] | 15 | Positive | Pt #8 |
| 8 | 46,XY,t(5;14)(q31;q32) | 13 | ND | Tono-oka et al, 1984 |
| 10 | 48,XX,+X,t(5;14)(q31;q32),+21c[8]/47,XX,+21c[12] | 40 | Positive | Pt #7 |
| 11 | 46,XY,t(5;14)(q31;q32)[4]/46,XY[12] | 25 | ND | Gallego et al, 2011 |
| 11 | t(5;14)(q31;q32) | NA | Positive | Kobayashi et al, 2018 |
| 11 | 46,XY,t(5;14)(q31;q32)[5]/46,XY[15] | 20 | Positive | Pt #1 |
| 13 | 46,XY[20] | 0 | ND | Yu et al, 2015 |
| 14 | 46,XY,t(5;14)(q31;q32)[3]/46,XY[24] | 11 | ND | Pt #4 |
| 14 | 46,XX,t(5;14)(q31;q32)[1]/46,XX[29] * | 3 | Failure | Pt #3 |
| 14 | 46,XY,t(5;14)(q31;q32)[4]/47,idem,+X[2]/46,XY[20] | 23 | Positive | Roberts et al, 2014 |
| 16 | 46,XY,t(5;14)(q31;q32)/46,idem,del(12)p(12) | NA | ND | Heerema et al, 1992 |
| 17 | 46,XY,t(5;14)(q31;q32),t(7;12)(p15;q13),+19,-20 | NA | ND | George et al, 2012 |
| 18 | 46,XY,t(5;14)(q31;q32)[2]/46,XY[18] | 10 | Positive | Pt #2 |
| 19 | 46,XY,t(5;14)(q?;q32)[22]/ 46,XY[3] | 88 | ND | Hogan et al, 1987 |
| 22 | 48,XY,+X,+4,t(5;14)(q31;q32),i(21)(q10)[7]/46,XY[32] | 18 | ND | Larsen et al, 2011 |
| 25 | 46,XY,t(5;14)(5pter→5q23::14q32→14qter; 14pter→14p12::5q31→5q23::14p12→14q32::5q31→5qter),  t(21;22)(q10;q10),+19/46,XY | NA | ND | McConnell et al, 1987 |
| 31 | 46,XY[25] | 0 | Positive | Pt #6 |
| 33 | 47,XY,+X,t(5;14)(q31;q32),i(7)(q10) | 20 | ND | Knuutila et al, 1993 |
| 39 | 46,XY,t(5;14)(q31;q32),der(16)t(3;16)(q21;p12)[20] | 100 | Positive | Pt #5 |
| 57 | 46,XX,t(5;14)(q31;q32) | NA | ND | Kaneko et al, 2014 |
| 60 | 46,XY,t(5;14)(q31;q32),del(15)(q2?3q26)[4]/46,XY[16] | 20 | ND | Toboso et al, 2017 |

­
