# Supplementary material for: B-ALL With t(5;14)(q31;q32); IGH-IL3 Rearrangement and Eosinophilia: A Comprehensive Analysis of a Peculiar IGH-Rearranged B-ALL
Source: Front Oncol. 2019 Dec 10;9:1374. doi: 10.3389/fonc.2019.01374 (PMC6914849; doi:10.3389/fonc.2019.01374)
Supplement: Supplementary file 3 [file Table_3.DOCX]

| Table S3. Recurrent gene deletions in the 6 newly reported patients with aCGH abnormalities | |
| --- | --- |
| Target genes of deletions | Incidence |
| *B-cell differentiation genes* |  |
| *IKZF1* | 5/6 |
| *PAX5* | 2/6 |
| *VPREB1* | 3/6 |
| *MEF2C* | 2/6 |
| *Other recurrent target genes* |  |
| *SLX4IP* | 4/6 |
| *ETV6* | 2/6 |
| *CDKN2A* | 3/6 |
| *ARHGAP24* | 2/6 |
| *ADD3* | 2/6 |
| *CD200-BTLA* | 2/6 |
