# Supplementary material for: B-ALL With t(5;14)(q31;q32); IGH-IL3 Rearrangement and Eosinophilia: A Comprehensive Analysis of a Peculiar IGH-Rearranged B-ALL
Source: Front Oncol. 2019 Dec 10;9:1374. doi: 10.3389/fonc.2019.01374 (PMC6914849; doi:10.3389/fonc.2019.01374)
Supplement: Supplementary file 4 [file Table_4.DOCX]

| **Table S4**. *IKZF1* status of 8 newly reported patients  *: aCGH performed at relapse only; **: abnormalities may have been unnoticed due to a low blast level (<20%).  NA: not available. NP: not performed  +: deleted  -: not deleted | | | |
| --- | --- | --- | --- |
|  | **a-CGH** | **MLPA** | **PCR for intragenic deletion** |
| **Pt #1** | + | NP | - |
| **Pt #2** | + | + | +  Exons 2-7 |
| **Pt #3** | -* | NP | - |
| **Pt #4** | -** | -** | -** |
| **Pt #5** | + | NP | +  Exons 4-7 |
| **Pt #6** | + | NP | - |
| **Pt #7** | + | + | NP |
| **Pt #8** | NA | NA | NA |
