# Supplementary figures and images for: B-ALL With t(5;14)(q31;q32); IGH-IL3 Rearrangement and Eosinophilia: A Comprehensive Analysis of a Peculiar IGH-Rearranged B-ALL
Source: Front Oncol. 2019 Dec 10;9:1374. doi: 10.3389/fonc.2019.01374 (PMC6914849; doi:10.3389/fonc.2019.01374)

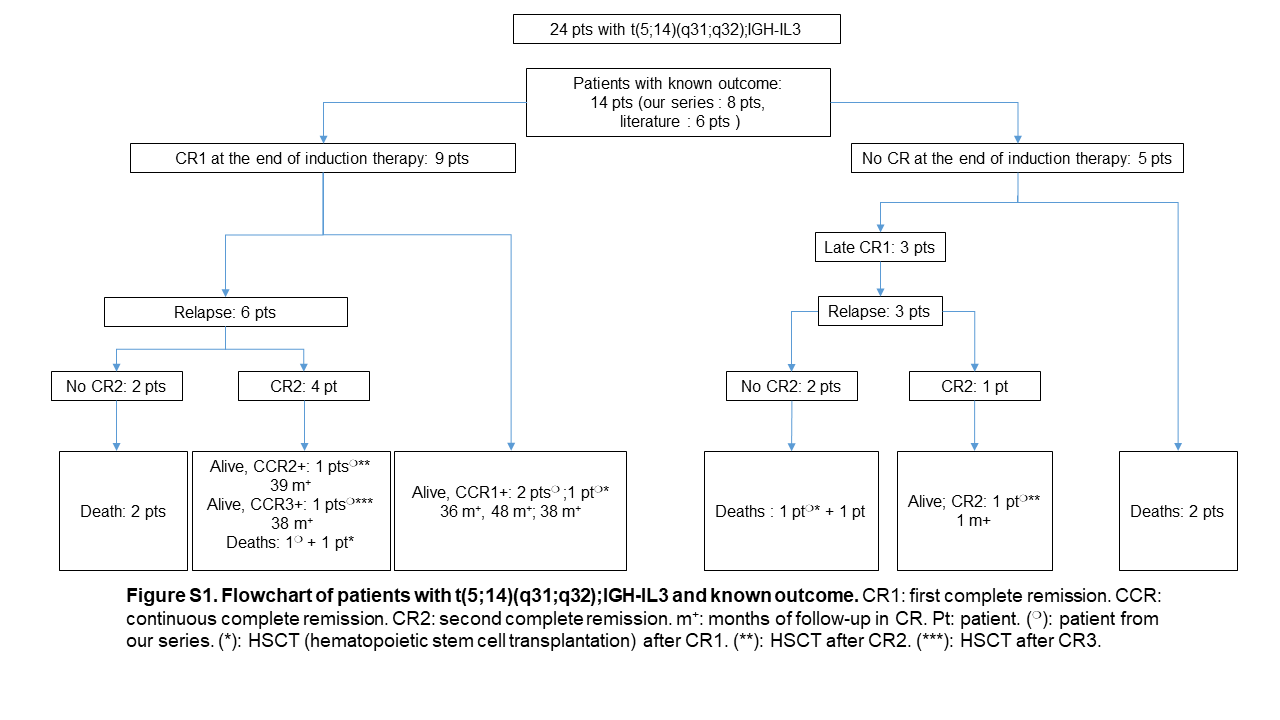

Supplement: Supplementary file 5 [file Image_1.TIF]
